# Mesenchymal stem cells deliver exogenous miR‐21 *via* exosomes to inhibit nucleus pulposus cell apoptosis and reduce intervertebral disc degeneration

**DOI:** 10.1111/jcmm.13316

**Published:** 2017-08-14

**Authors:** Xiaofei Cheng, Guoying Zhang, Liang Zhang, Ying Hu, Kai Zhang, Xiaojiang Sun, Changqing Zhao, Hua Li, Yan Michael Li, Jie Zhao

**Affiliations:** ^1^ Department of Orthopaedic Surgery Shanghai Key Laboratory of Orthopaedic Implants Shanghai Ninth People's Hospital Shanghai JiaoTong University School of Medicine Shanghai China; ^2^ Department of Neurosurgery University of Rochester School of Medicine and Dentistry Rochester NY USA; ^3^ Department of Orthopedics The General Hospital of Chinese People's Liberation Army Beijing China; ^4^ Department of Orthopedics Subei People's Hospital of Jiangsu Province Clinical Medical College of Yangzhou University Yangzhou Jiangsu China; ^5^ Department of Toxicity Evaluation Shanghai Municipal Center for Disease Control and Prevention Shanghai China

**Keywords:** mesenchymal stem cells, exosomes, intervertebral disc degeneration, nucleus pulposus cells, apoptosis, miR‐21, phosphatase and tensin homolog

## Abstract

Although mesenchymal stem cells (MSCs) transplantation into the IVD (intervertebral disc) may be beneficial in inhibiting apoptosis of nucleus pulposus cells (NPCs) and alleviating IVD degeneration, the underlying mechanism of this therapeutic process has not been fully explained. The purpose of this study was to explore the protective effect of MSC‐derived exosomes (MSC‐exosomes) on NPC apoptosis and IVD degeneration and investigate the regulatory effect of miRNAs in MSC‐exosomes and associated mechanisms for NPC apoptosis. MSC‐exosomes were isolated from MSC medium, and its anti‐apoptotic effect was assessed in a cell and rat model. The down‐regulated miRNAs in apoptotic NPCs were identified, and their contents in MSC‐exosomes were detected. The target genes of eligible miRNAs and possible downstream pathway were investigated. Purified MSC‐exosomes were taken up by NPCs and suppressed NPC apoptosis. The levels of miR‐21 were down‐regulated in apoptotic NPCs while MSC‐exosomes were enriched in miR‐21. The exosomal miR‐21 could be transferred into NPCs and alleviated TNF‐α induced NPC apoptosis by targeting phosphatase and tensin homolog (PTEN) through phosphatidylinositol 3‐kinase (PI3K)‐Akt pathway. Intradiscal injection of MSC‐exosomes alleviated the NPC apoptosis and IVD degeneration in the rat model. In conclusion, MSC‐derived exosomes prevent NPCs from apoptotic process and alleviate IVD degeneration, at least partly, *via* miR‐21 contained in exosomes. Exosomal miR‐21 restrains PTEN and thus activates PI3K/Akt pathway in apoptotic NPCs. Our work confers a promising therapeutic strategy for IVD degeneration.

## Introduction

Low back pain is a highly prevalent condition and the leading cause of years lived with disability worldwide 1. A widely recognized contributor to low back pain is intervertebral disc (IVD) degeneration, which is the major cause of a series of lumbar degenerative disc diseases. IVD comprises the inner nucleus pulposus (NP) and surrounded annulus fibrosus (AF). The NP is a hydrated gelatinous tissue and the major functional composition of the IVD to confront diverse external mechanical stimuli. Nucleus pulposus cells (NPCs) are the main type of cells residing in the NP and responsible for synthesizing and maintaining the gelatinous extracellular matrix. IVD degeneration is characterized by loss of NPCs and extracellular matrix. Increased levels of inflammatory cytokines such as tumour necrosis factor‐a (TNF‐α) have been observed in degenerated IVD tissue and proven to induce excessive NPC apoptosis and resultant IVD degeneration 2, 3, 4, 5, 6. Thus, it is necessary to find an effective way to inhibit NPC apoptosis triggered by inflammatory cytokines.

There has been great interest in utilizing MSCs transplantation as a potentially effective strategy for the treatment of IVD degeneration 7, 8. The effects of transplanted MSCs are partly because of their ability to mitigate NPC apoptosis 9. However, the underlying mechanism of this therapeutic process has not been fully explained 10. The direct differentiation of MSCs and the interaction between MSCs and NPCs with direct cell contact have been implicated in the therapeutic benefits 11, 12, 13. Meanwhile, emerging evidence has suggested the role of indirect paracrine effect by MSCs in stimulating intrinsic reparative potential of NPCs 14, 15. A recent study has found extensive transfer of vesicles between MSCs and NPCs 16. Nevertheless, what these vesicles contain, and whether their delivery is a potentially mechanism underlying MSC‐induced repair of NPCs remain elusive.

Recently, accumulating evidence has demonstrated that MSCs released a type of specialized extracellular vesicles termed exosomes to provide therapeutic benefits 17. Exosomes are membranous nano‐sized vesicles secreted by a variety of cells, merging their membrane contents into the recipient cell membrane and transferring factors into the recipient cells. They have been increasingly reported as the principal therapeutic agent mediating MSCs paracrine action that underpins the therapeutic capabilities of MSCs in inhibiting apoptosis, reducing injury and/or promoting repair in the recipient cells 18. Thus, MSC‐secreted exosomes (MSC‐exosomes) are likely implicated in regulating apoptosis in NPCs. MSC‐exosomes carry a complex cargo of nucleic acids, proteins and lipids, with abundant miRNAs 19, and transfer these contents into the recipient cells, rationalizing potential applications of MSC‐exosomes as an alternative, cell‐free therapy 20. These exosomes‐delivered effectors, particularly miRNAs, can provide an effective therapeutic method of attenuating the recipient cell apoptosis 21, 22. miRNAs have considerable potential to be a research focus for prevention and treatment of IVD degeneration, especially for targeting promotion or suppression of NPC apoptosis 5, 23, 24, 25, 26, 27, 28. As a possible mediator of MSC‐exosomes and NPC apoptosis, exosomal miRNAs have therapeutic potential for NPC apoptosis and deserve to be further explored. Here, we explored the protective effect of MSC‐exosomes on NPCs in a cell and rat model, and specifically investigated the regulatory effect of miRNAs in MSC‐exosomes and associated mechanisms for NPC apoptosis.

## Materials and methods

### Cell isolation and culture

Human bone marrow aspirates and NP tissue samples were obtained from five separate patients (age 32.8 ± 8.3) undergoing spine surgery because of burst thoracolumbar fracture to insure that the results were not donor specific (Table 1). The experimental protocol was approved by the institutional review board with informed consent from the patients. The NP tissues were treated with 0.25% pronase (Sigma‐Aldrich, Louis, MO, USA) for 30 min. and 0.2% collagenase type II (Invitrogen, Carlsbad, CA, USA) for 4 hrs at 37°C. The digest was filtered through a 70‐μm pore size mesh and then cultured in Dulbecco's modified Eagle's medium (DMEM; Gibco, Grand Island, NY, USA) with 10% foetal bovine serum (FBS; Invitrogen), 1% penicillin–streptomycin (Sigma‐Aldrich), 2 mM glutamine (Sigma‐Aldrich) and 50 μg/mL L‐ascorbic acid (Sigma‐Aldrich) in T25 flasks at 37°C in 5% CO_2_. When grew to confluence, the cells were digested by 0.25% trypsin/1 mM EDTA and passed into bigger flasks for expansion. The NPCs from passage 4 or 5 were plated into experimental plates for all of the experiments. MSCs were isolated by density gradient centrifugation and adherence to tissue culture plastic. Cells were expanded in alpha‐minimum essential medium (α‐MEM, Gibco) containing 10% FBS and 1% penicillin–streptomycin. MSCs from passages 3 or 4 were used. Two independent MSC populations (2 different donors) were randomly selected and characterized by the expression of CD73, CD90 and CD105 and lack of expression of CD34 and CD45 using flow cytometry (Fig. S1). To obtain conditioned medium (CM), the medium was collected after 48 hrs of incubation with cells and centrifuged at 2000 *g* for 10 min. at 4°C. Supernatant was filtered through a 0.22‐μm filter to thoroughly remove the cellular debris.

**Table 1:** MSCs and NPCs donors used for this study

Donor ID	Sex	Age	Site of fracture	Site of aspiration (vertebral body) for MSCs	Site of dissection (intervertebral disc) for NPCs
#1	Male	45	L1	L1/L2	T12/L1, L1/L2
#2	Female	29	L2	L1/L2	L1/L2, L2/3
#3	Male	31	T12	T11/T12/L1	T11/T12, T12/L1
#4	Male	36	L1	T12/L1/L2	T12/L1, L1/L2
#5	Male	23	L1	L1/L2	T12/L1, L1/L2

MSCs, mesenchymal stem cells; NPCs, nucleus pulposus cells; T, thoracic vertebral body; L, lumbar vertebral body.

The donors were all patients undergoing vertebral resection because of burst thoracolumbar fracture, and healthy without metabolic disease, inherited illnesses, or other diseases that may affect the current study. MSCs and NPCs were obtained from bone marrow aspirates of the vertebral body and dissection of the intervertebral disc tissues, respectively.

### Exosomes isolation

MSC‐exosomes isolation procedures were performed as previously described with some modifications 29. Briefly, MSCs were switched to OptiMEM (Invitrogen) and conditioned for 48 hrs before exosomes isolation. Culture supernatant was harvested by centrifugation at 300 *g* for 10 min. at 4°C to eliminate cells, at 2000 *g* for 20 min. to obtain apoptotic body and at 20,000 g for 30 min. to obtain microvesicles. At each step, the supernatant was transferred to new tubes and the pellets were immediately resuspended in phosphate‐buffered saline (PBS). The remaining supernatant was filtrated through a 0.22‐μm filter to remove particles larger than 200 nm, followed by ultracentrifugation using the Optima L‐100xp ultracentrifuge (Beckman Coulter, Brea, CA, USA) at 120,000 *g* for 2 hrs at 4°C. The pellets were washed with PBS, ultracentrifuged again and resuspended in PBS. Exosomes were pooled for experiments or stored at −80°C. As a control, we also obtained CM and exosomes from normal human fibroblasts (Stem Cell Bank, Chinese Academy of Sciences, Shanghai, China).

### Exosomes characterization

MSC‐derived particles were resuspended and further diluted in 1 ml PBS to analyze their number and size distribution using the NanoSight NS300 system (Malvern, UK) according to manufacturer's protocol. The particle morphology was observed using transmission electron microscope (TEM). Resuspended 5 μl of sample was dropped onto formvar carbon‐coated 200‐mesh grids to incubate for 10 min., fixed using 2.5% glutaraldehyde for 5 min. and stained with 2% uranyl acetate for 1 min. The grids were examined using the H‐7650 TEM (Hitachi, Tokyo, Japan) at 80 kV. These particles were detected based on the markers expression of exosomes (Alix, TSG101, CD9, CD63) and MSCs (CD105) using Western blot.

### Exosomes uptake by NPCs

Purified MSC‐exosomes were incubated with PKH26 (Sigma‐Aldrich) for 5 min. at room temperature. After being washed twice in PBS with 120,000 *g* centrifugation for 90 min., the labelled exosomes were suspended in basal medium and incubated with NPCs for 12 hrs at 37°C. NPCs were washed twice with PBS. To stain the nuclei, 4′,6‐diamidino‐2‐phenylindole (DAPI; Sigma‐Aldrich) was added for 10 min. The stained cells were observed under the IX53 fluorescence microscope (Olympus, Tokyo, Japan).

### Flow cytometry analysis

NPCs were plated into 6‐well plates at a density of 1 × 10^5^ cells per well. To test the apoptosis‐inducing effect of TNF‐α on cells, NPCs were treated with 0, 1, 5, 10 or 30 ng/ml TNF‐α (Sigma‐Aldrich) for 12 hrs. To assess the anti‐apoptotic effect of MSC‐CM or MSC‐exosomes, the basic medium of NPCs was replaced by MSC‐CM, CM from TNF (5 ng/ml) pretreated MSC (TNF‐MSC), exosome‐depleted fraction of MSC‐CM or fibroblast‐CM, or supplemented with exosomes (1 μg/ml) from MSC, TNF‐MSC or fibroblast for 36 hrs. Cells kept in the basic medium served as controls. All cells were then treated with TNF‐α (5 ng/ml) for 12 hrs. Following treatment, NPC apoptosis rates were evaluated by flow cytometry using an Annexin V/PI apoptosis detection kit (BD Biosciences, Franklin Lakes, NJ, USA). NPCs were washed twice with PBS, resuspended in binding buffer and incubated with 5 μl FITC‐Annexin V and 5 μl PI for 15 min. at room temperature. Staining cells were analysed using the FACScan flow cytometry system (Becton Dickinson, San Diego, CA, USA).

### microRNA array hybridization and data analysis

NPCs from three randomly selected samples (5 × 10^6^ per sample) with and without treatment of TNF‐α were harvested as the test and reference sample, respectively. Total RNA was labelled using the miRCURY LNA microRNA Hi‐Power Labeling Kit, Hy3/Hy5, and hybridized on the miRCURY LNA microRNA Array (Exiqon, Vedbæk, Denmark). Three independent hybridizations for each sample were performed on chip. After hybridization, the microarray slides were scanned using GenePix 4000B scanner (Axon, Foster City, CA, USA) and the data were analysed using GenePix Pro V6.0 software (Axon). Expression data were normalized using the locally weighted scatter plot smoothing (LOWESS) regression algorithm. miRNAs with twofold difference and statistical significance between groups were considered as differentially expressed. Hierarchical cluster and heat map analyses were performed using the MultiExperiment Viewer v4.8 program of TM4 Microarray Software Suite. The DIANA‐mirPath v3.0 database was used to identify target genes and pathways potentially altered by the down‐regulated miRNAs in TNF‐α‐treated NPCs. The software created a clustering of the selected miRNAs based on their influence on molecular pathways 30.

### RNA extraction and quantification

Total RNAs from cells/tissues and exosomes were extracted using miRNeasy Mini kit (Qiagen, Venlo, The Netherlands) and SeraMir kit (System Biosciences, Mountain View, CA, USA), respectively. miRNA levels were determined using stem‐loop miRNA RT‐PCR Quantitation kit (GenePharma, Shanghai, China). Specific miRNA primers were synthesized by GenePharma. RNA was reverse‐transcribed using PrimeScipt RT Reagent Kit (Takara, Kusatsu, Japan). cDNA was used to perform quantitative reverse transcription‐PCR (qRT‐PCR) on the 7500 Sequence Detection System (ABI, Foster City, CA, USA) using SYBR Premix Ex Taq (Takara). Fold changes in expression were calculated by a comparative threshold cycle (Ct) method using the formula 2^−(ΔΔct)^. U6 and cel‐mir‐39 (GenePharma) were used as controls for miRNA in cells/tissues and exosomes, respectively, and GAPDH was used as a control for mRNA in cells.

### Exosomal miRNA degradation

MSC‐exosomes were suspended in PBS with 5% Triton, treated with 0.4 mg/ml RNase A for 10 min. at 37°C, and then treated with 0.1 mg/ml Proteinase K for 20 min. at 37°C. The levels of miR‐21 were measured by qRT‐PCR.

### Protein analysis

Exosomes, cells or tissues were lysed, and their protein contents were measured using the Micro BCA Protein Assay Kit (Thermo, Waltham, MA, USA). The lysates were centrifuged, subjected to sodium dodecyl sulphate‐polyacrylamide gel electrophoresis gels and transferred to polyvinylidene fluoride membranes. The membranes were blocked and incubated with the specific primary antibody overnight at 4°C and horseradish peroxidase‐labelled secondary antibodies (dilution 1:5000–10,000; Abcam, Cambridge, MA, USA) were added. Protein expression was visualized using enhanced chemiluminescence reagents (Amersham, Piscataway, NJ, USA) and the ChemiDoc XRS system (Bio‐Rad, Hercules, CA, USA). The primary antibodies used are as follows: CD9 (#ab92726, 1:2000; Abcam), CD63 (#ab134045, 1:2000; Abcam), Alix(#ab117600, 1:2000; Abcam), TSG101(#ab30871, 1:1000; Abcam), CD105(#ab107595, 1:500; Abcam), phosphatase and tensin homolog (PTEN; #9559, 1:1000; Cell Signaling Technology, CST, Beverly, MA, USA), phospho‐PTEN (#9549, 1:1000; CST), Akt (#2920, 1:2000; CST), phospho‐Akt (#4058, 1:1000; CST), Bad (#9268, 1:1000; CST), Bcl‐2 (#3498, 1:1000; CST), Bax (#5023, 1:1000; CST), caspase‐3 (#9665, 1:1000; CST) and cleaved caspase‐3 (#9664, 1:1000; CST). The antibody for GAPDH (#9485, 1:2500; Abcam) was used as a control.

### Transfection

NPCs (2 × 10^5^/well) were seeded in 6‐well plates at 24 hrs before transfection. The synthesized miR‐21 agonist (agomir‐21), negative control of agomir (agomir‐NC), miR‐21 antagonist (antagomir‐21), negative control of antagomir (antagomir‐NC), PTEN siRNA or scramble siRNA (GenePharma; Table S1) was transfected using OptiMEM and Lipofectamine3000 (Invitrogen) according to the manufacturer's instructions. Transfected efficiencies were assessed by qRT‐PCR. Cells were incubated for 48 hrs or for 36 hrs before adding TNF‐α (5 ng/ml) for an additional 12 hrs. In some experiments, cells were pretreated with a phosphoinositide 3‐kinase (PI3K) inhibitor (LY294002, 10 μM; Sigma‐Aldrich) for 1 hr.

### Luciferase reporter assay

The human 3′‐UTR of PTEN gene was amplified by PCR using specific primers (Table S1). Either wild‐type or mutant PTEN‐3′UTR fragment was inserted into the Xba1 restriction sites of pmirGLO luciferase vector (Promega, Madison, WI, USA). NPCs were plated in 96‐well plates at 8 × 10^3^ cells per well and cotransfected with 0.2 μg pmirGLO‐PTEN‐3′UTR reporter plasmid and 80 nM agomir‐21 or agomir‐NC using Opti‐MEM and Lipofectamine 3000. The luciferase activity was measured using the Dual‐Luciferase Reporter Assay System (Promega) after 48 hrs. The optical density of the resulting solution was measured using the SpectraMax i3x multiplate reader (Molecular Devices, Sunnyvale, CA, USA). The ratio of firefly and Renilla luciferase activity was calculated.

### Determination of cell proliferation

Cell proliferation was evaluated using the cell counting Kit‐8 (CCK‐8; Dojindo, Kumamoto, Japan). NPCs were seeded in 96‐well plates at 5 × 10^3^ cells per well after transfection. The plates were incubated for 1, 2, 3 or 4 days at 37°C. Plates were read on the SpectraMax i3x reader at 450 nm.

### Intradiscal injection of exosomes in a rat model of IVD degeneration

A total of 42 male Sprague–Dawley rats, aged 3 months, were used for the experiments *in vivo*. A model of IVD degeneration established by Zhang *et al*. 31 was adopted. Thirty‐six rats underwent the surgery while six rats underwent no surgical intervention as negative controls. The rats were placed in a prone position after anaesthesia with an intraperitoneal injection of 90 mg/kg ketamine and 10 mg/kg xylazine. Under fluoroscopic guidance, three IVDs (Co6/7, Co8/9 and Co10/11) were punctured by a 20‐gauge needle from the dorsal side. The needle punctured through the centre of the disc until the opposite side, rotated 180° and held for 10 sec. Co7/8 and Co9/10 were left intact as self‐controls. After the surgery, the wound was covered with gauze and a standard postoperative procedure was performed. One week after the initial operation, the rats were randomly divided into six groups (non‐injection, normal saline, MSC‐exosomes, fibroblast‐exosomes, antagomir‐21 MSC‐exosomes and antagomir‐NC MSC‐exosomes group) with six rats in each group. After anaesthesia, a small incision was made to expose the previous punctured IVDs from the left side. A total of 2 μl sterile saline containing different purified exosomes (approximately 1.5 × 10^6^ particles) or not was slowly injected into the punctured discs using a 33‐gauge needle (Hamilton, Bonaduz, Switzerland) attached to a microlitre syringe (Hamilton). The injection procedure was repeated after 4 weeks.

### Radiography and MRI examination

All rats underwent radiography immediately before the IVD puncture and two injections, and 4 weeks after the second injection. Disc height was measured using the ImageJ software (US National Institutes of Health) and expressed as the disc height index (DHI) using the method as previously described 32. Changes in the DHI of the punctured IVDs were expressed as %DHI (%DHI = post‐punctured DHI/pre‐punctured DHI × 100%). After the last radiography, MRI was performed on all rats using a 7.0 T animal specific MRI system (Bruker Pharmascan, Ettlingen, Germany). T2‐weighted sections in the median sagittal plane were obtained using the following settings: a fast spin echo (SE) sequence with a time to repetition (TR) of 3000 ms and a time to echo (TE) of 70 ms; the slice thickness was 0.5 mm with a 0 mm gap. The Pfirrmann classification was used to assess the degree of IVD degeneration 33. The average score of the punctured IVDs was calculated as the degeneration grade of each rat.

### Histological evaluation and *in situ* apoptosis detection

After the MRI examination, all rats were sacrificed by intraperitoneal administration of overdose pentobarbital sodium. The target levels were harvested *en bloc* and Co6/7 and Co10/11 NP samples were obtained to perform qRT‐PCR and Western blot analysis, respectively, using the aforementioned methods. Co8/9 discs were fixed in 4% paraformaldehyde for 48 hrs, decalcified in 25% formic acid and 10% sodium citrate for 2 days, embedded in paraffin, and sectioned (5 μm) along the midsagittal plane. Sections were used for haematoxylin–eosin (HE) or terminal deoxynucleotidyl transferase (TdT)‐mediated dUTP nick end labelling (TUNEL) staining. Histological images were analysed using the BX53 microscope (Olympus). The grading score of HE staining was made as per the criteria established by Masuda *et al*. 32 Apoptotic activity was detected using the *In Situ* Cell Death Detection Kit Fluorescein (Roche, Mannheim, Germany) according to the manufacturer's instructions. Sections were overlaid with the Vectashield Hard Set mounting medium (VECTOR Laboratories, Burlingame, CA, USA) containing DAPI. Images were captured using filters for fluorescein isothiocyanate (FITC) and DAPI. Total and TUNEL‐positive cells were counted in three fields in the NP region using the Image‐Pro Plus software (Media Cybernetics, Silver Spring, MA, USA) and summed up. The percentage of TUNEL positive cells relative to total cells was calculated. All image assessments were performed by two independent blind observers.

### Statistical analysis

All data were expressed as mean ± S.D. from six separate experiments, unless otherwise noted. Data distribution was tested with Shapiro–Wilk test. The equality of variances was tested with Levene test. Statistical analysis was performed by unpaired two‐tailed Student's *t*‐test (normal distribution and equal variances), Welch *t*′‐test (unequal variances) or Mann–Whitney *U*‐test (non‐normal distribution) between two groups. Multiple group comparisons were performed by one‐way analysis of variance (normal distribution) or Kruskal–Wallis (non‐normal distribution) test followed by Bonferroni's or Dunn *post hoc* test. All statistical analyses were performed with Prism version 7.0 (GraphPad Software, La Jolla, CA, USA) or SPSS software version 19.0 (IBM, Armonk, NY, USA). For all analyses, group differences were considered statistically different for *P* < 0.05 between groups.

## Results

### MSC‐CM inhibited TNF‐α induced NPC apoptosis

**Figure 1:**
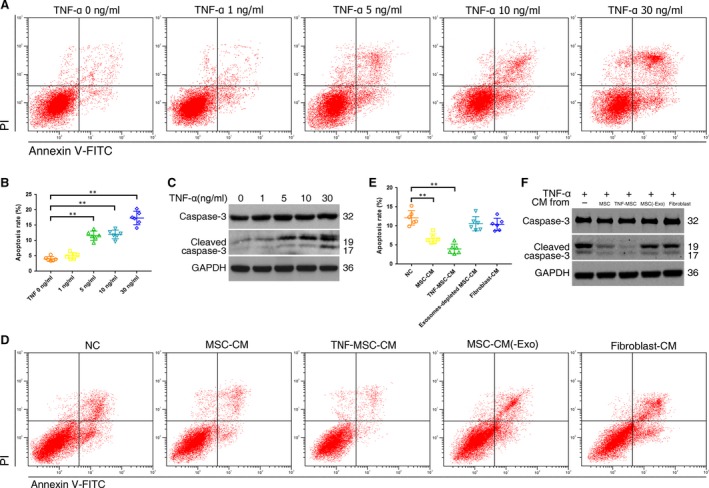
Effect of TNF‐α on NPC apoptosis and anti‐apoptotic function of MSC‐conditioned medium (CM). (**A**) NPCs were treated with 0, 1, 5, 10 or 30 ng/ml of TNF‐α for 12 hrs. Representative dot plots of apoptosis flow cytometry detection were shown after Annexin V‐FITC/propidium iodide (PI) dual staining. (**B**) The percentage of the apoptotic cells was 4.0% ± 0.7% (0 ng/ml), 5.1% ± 1.0% (1 ng/ml), 11.6% ± 1.5% (5 ng/ml), 12.0% ± 1.4% (10 ng/ml) and 17.4% ± 2.2% (30 ng/ml) (** *P <* 0.01 by Kruskal–Wallis test). (**C**) Western blot analysed caspase‐3 and cleaved caspase‐3 in NPCs after treatment of different TNF‐α concentrations. (**D**) Anti‐apoptotic activities of MSC‐CM, CM of TNF‐α (5 ng/ml) pretreated MSCs (TNF‐MSC‐CM), exosomes‐depleted MSC‐CM (MSC‐CM(‐Exo)) and fibroblast‐CM were assessed on TNF‐α‐treated NPCs. Representative dot plots of cell apoptosis were shown. (**E**) MSC‐CM and TNF‐MSC‐CM reduced NPC apoptosis rates from 12.1% ± 1.9% to 6.7% ± 1.2% and 4.0% ± 1.3%, respectively (** *P <* 0.01 by Kruskal–Wallis test). Neither exosomes‐depleted MSC‐CM nor fibroblast‐CM remarkably reduced the number of dead cells (10.6% ± 1.9%; 10.4% ± 1.6%). (**F**) Western blot analysed caspase‐3 and cleaved caspase‐3 in NPCs after treatment of different CMs.

Apoptosis of NPCs treated with different concentrations of TNF‐α was measured using flow cytometry, and activation of apoptotic pathways was detected by assessment of cleaved caspase‐3 using Western blot. The results demonstrated that the lowest dose of TNF‐α causing NPC apoptosis was 5 ng/ml. After that, the apoptosis of NPCs gradually increased with the increase of TNF‐α concentrations (Fig. 1A and B). Analogously, the expression levels of cleaved caspase‐3 in NPCs increased following 5 ng/ml TNF‐α treatment (Fig. 1C). Anti‐apoptotic activities of MSC‐CM were assessed in NPCs. MSC‐CM and TNF‐MSC‐CM both reduced dead cells compared with the control. Neither exosomes‐depleted MSC‐CM nor fibroblast‐CM reduced the number of dead cells (Fig. 1D and E). Similar changes were found in cells positive for activated caspase‐3 (Fig. 1F).

### MSC‐exosomes were taken up by NPCs and suppressed NPC apoptosis

**Figure 2:**
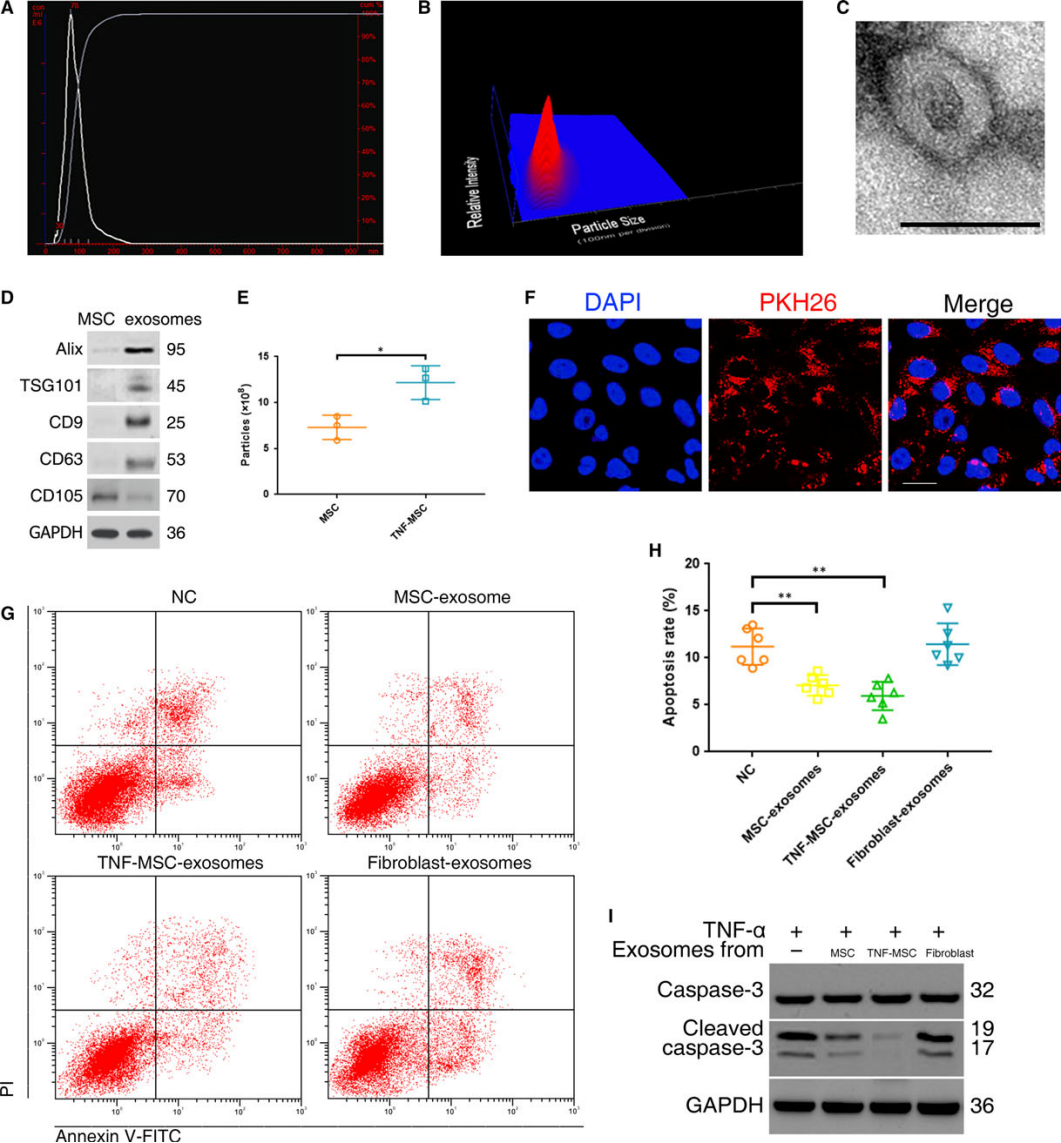
Exosomes secreted by MSCs were taken up by NPCs and suppressed NPC apoptosis. (**A**) Purified particles were analysed by nanoparticle tracking analysis (NTA). Sizes of the particles were between 30 and 200 nm. Modal and mean size was 78 nm and 87 nm, respectively. Concentration was 6.85 × 10^8^ particles/ml. (**B**) 3D heat map of the size distribution of the particles. (**C**) Transmission electron micrograph of purified particles. The image showed small vesicles of approximately 100 nm in diameter. Scale bar = 100 nm. (**D**) Expression of exosomes markers (ALIX, TSG101, CD9, CD63) and MSC marker (CD105) detected by Western blot. The protein expression of exosomes markers was detectable in MSC‐exosomes but not MSCs. (**E**) NTA counted the production of particles derived from MSCs and those from MSCs pretreated with 5 ng/ml TNF‐α (TNF‐MSC) (*n* = 3; *P < 0.05 by *t*‐test). (**F**) PKH26‐labelled MSC‐exosomes showed red fluorescence in the cytoplasm of NPCs. Scale bar = 25 μm. (**G**) Anti‐apoptotic activities of MSC‐exosomes, exosome secreted by TNF‐MSC (TNF‐MSC‐exosomes) and fibroblast‐exosomes were assessed on TNF‐α‐treated NPCs. Representative dot plots of apoptosis flow cytometry detection were shown. (**H**) The percentage of apoptotic NPCs could be decreased to 7.1% ± 1.1% by MSC‐exosomes or to 6.0% ± 1.5% by TNF‐MSC‐exosomes compared with the control (11.2% ± 1.9%), while fibroblast‐derived exosomes did not cause significant change of apoptosis rate (11.5% ± 2.2%) (**P < 0.01 by Kruskal–Wallis test). (**I**) Western blot analysed caspase‐3 and cleaved caspase‐3 in NPCs after treatment of different exosomes.

The morphology and phenotypes of isolated exosomes were identified as per the characteristics of exosomes. The modal size of MSC‐derived particles was 78 ± 8 nm, corresponding to the expected size of exosomes, and the concentration was 7.31 ± 1.32 × 10^8^ particles/ml (Fig. 2A and B). The particles were revealed as cup‐shaped vesicles with double‐layer membrane structure and <100 nm in diameter using TEM (Fig. 2C). The protein expression of exosomes markers (Alix, TSG‐101, CD9 and CD63) and CD105 were all detectable in the particles (Fig. 2D). The above properties analysis identified these collected particles as MSC‐derived exosomes. To observe the effect of TNF‐α on exosomes secretion of MSCs, exosomes were collected from MSCs pretreated with 5 ng/ml TNF‐α for 12 hrs, and exosomes concentrations were analyzed using nanoparticle trafficking analysis. The results showed that the exosomes concentrations increased from 7.31 ± 1.32 × 10^8^ particles/ml to 12.17 ± 1.83 × 10^8^ particles/ml after the pretreatment (Fig. 2E), suggesting that TNF‐α could promote the production of MSC‐exosomes. After incubated with NPCs, PKH26‐labelled exosomes showed red fluorescence in the cytoplasm of NPCs (Fig. 2F), indicating exosomes uptake by NPCs. The percentage of TNF‐α‐induced apoptotic NPCs could be decreased by MSC‐exosomes or TNF‐MSC‐exosomes, but not fibroblast‐exosomes (Fig. 2G and H). Accordingly, the increases of activated caspase‐3 were suppressed after the cells were pretreated with MSC‐exosomes, but not fibroblast‐exosomes (Fig. 2I). These results demonstrated that exosomes were the active component of MSC‐CM, and that functional activities of exosomes depended on the parent cell type.

### MSC‐exosomes were enriched in miR‐21, which could be transferred into NPCs

**Figure 3:**
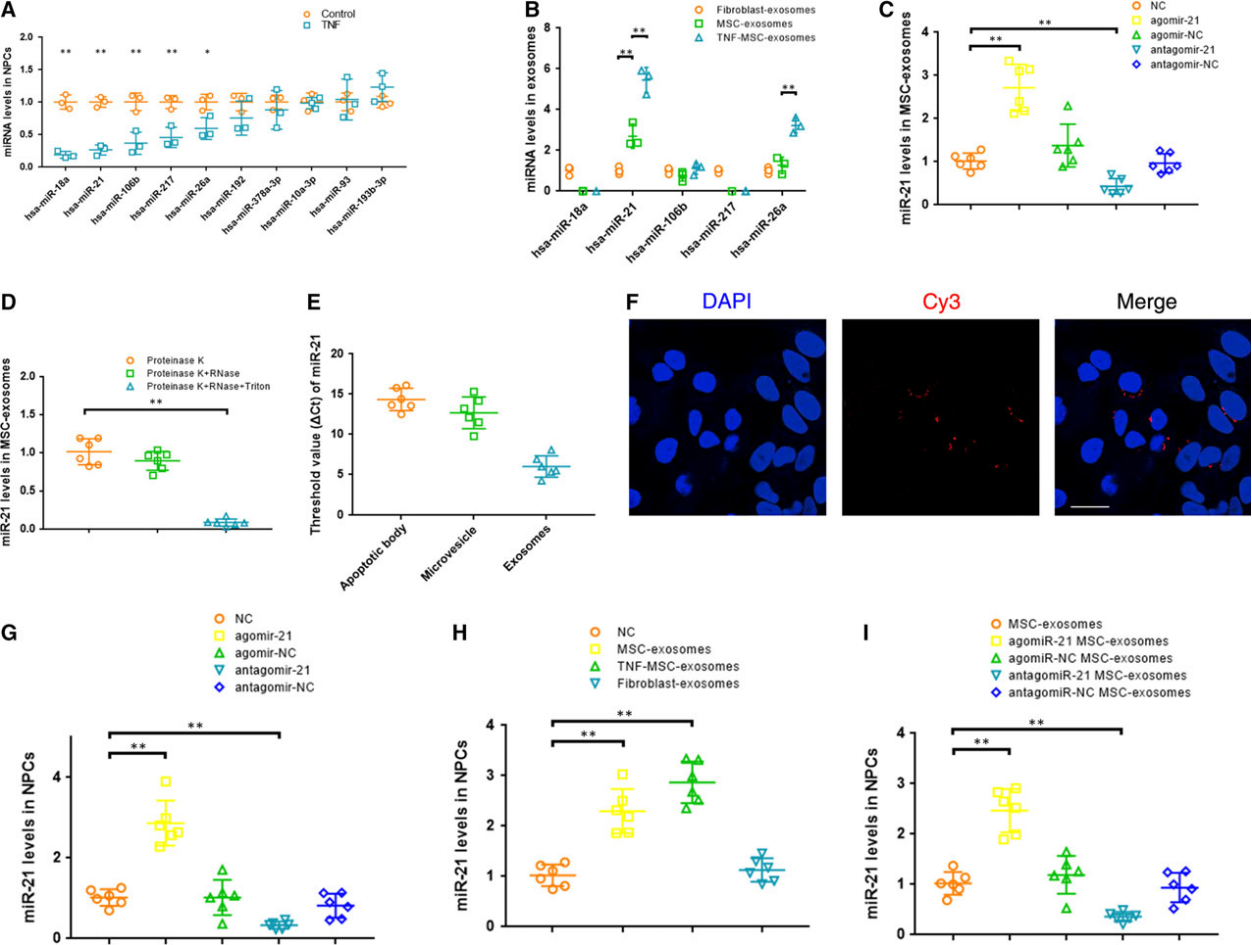
MSC‐exosomes were enriched in miR‐21, which could be transferred into NPCs (**A**) qRT‐PCR revealed that five miRNAs were significantly down‐regulated in TNF‐α‐treated NPCs compared with non‐treated cells (*n* = 3; *P < 0.05, **P < 0.01 *versus* the control by *t*‐test, *t*′‐test or Mann–Whitney *U*‐test). (**B**) qRT‐PCR showed that miR‐21 contents in MSC‐exosomes were significantly greater than that in fibroblast‐derived exosomes (*n* = 3; **P < 0.01 by anova or Kruskal–Wallis test). (**C**) Overexpression or knockdown of miR‐21 was confirmed by qRT‐PCR in exosomes derived from MSCs transfected with a miR‐21 agonist or antagonist (**P < 0.01 by Kruskal–Wallis test). (**D**) qRT‐PCR showed that miR‐21 within lipid‐bilayered exosomes was protected from RNase degradation (**P < 0.01 by anova test). (**E**) Quantitative analysis of miR‐21 was performed by qRT‐PCR on the pellets of extracellular vesicles isolated from MSC supernatant by differential centrifugation. (**F**) Exosomes were collected from the supernatant of MSCs transfected with cy3‐labeled agomir‐21. NPCs incubated with these exosomes exhibited a granular fluorescent pattern within the cytoplasm. Scale bar = 25 μm (**G**) qRT‐PCR showed that miR‐21 levels in NPCs were up‐regulated by the miR‐21 agonist and down‐regulated by the miR‐21 antagonist (**P < 0.01 by Kruskal–Wallis test). (**H**) qRT‐PCR showed that the decreased levels of miR‐21 in TNF‐α‐treated NPCs were rescued by incubation with MSC‐exosomes or exosomes from TNF‐α (5 ng/ml) pretreated MSCs (TNF‐MSC‐exosomes) but not with fibroblast‐exosomes (**P < 0.01 by Kruskal–Wallis test). (**I**) qRT‐PCR showed that overexpression or knockdown of intracellular miR‐21 levels was confirmed on MSC‐exosomes treated apoptotic NPCs when exposed to miR‐21‐overexpressing or miR‐21‐deficient exosomes (**P < 0.01 by Kruskal–Wallis test).

A microarray chip was used to compare miRNA levels in TNF‐α‐treated NPCs to those of untreated cells. Totally 2672 capture probes were detected. Among the 19 differentially expressed miRNAs, nine were up‐regulated while 10 were down‐regulated (Table S2, Fig. S2). The levels of the 10 down‐regulated miRNAs were further analysed by qRT‐PCR. Five miRNAs (miR‐18a, miR‐21, miR‐106b, miR‐217 and miR‐26a) were significantly down‐regulated in TNF‐α‐treated NPCs compared with non‐treated cells (Fig. 3A). We investigated the levels of the 5 miRNAs in exosomes, only miR‐21 contents in MSC‐exosomes were significantly greater than those in fibroblast‐exosomes (Fig. 3B). After pretreated with TNF‐α, MSC secreted exosomes exhibiting elevated intra‐exosomal miR‐21 concentration. To investigate the contribution of miR‐21 to the MSC‐exosomes effect, we developed miR‐21‐overexpressing or miR‐21‐deficient exosomes by transfecting MSCs with a miR‐21 agonist or antagonist followed by exosomes isolation. Successful overexpression or knockdown of miR‐21 was confirmed by qRT‐PCR on resultant exosomes (Fig. 3C). MSC‐exosomes was exposed to RNase A in the absence or presence of triton, with proteinase K added to dissociate protein complexes that may shield RNA. The results indicated that miR‐21 within lipid‐bilayered exosomes was protected from RNase degradation (Fig. 3D). Quantitative analysis of miR‐21 was performed on the pellets of extracellular vesicles generated by differential centrifugation. According to the ΔCt value, miR‐21 mainly existed in exosomes but not in apoptotic body or microvesicle isolated from MSC supernatant (Fig. 3E). Exosomes were collected from the supernatant of MSCs transfected with cy3‐labeled agomir‐21. NPCs incubated with these exosomes exhibited a granular fluorescent pattern within the cytoplasm (Fig. 3F), indicating the incorporation of exosomal miR‐21 into NPCs.

To explore the role of miR‐21 in TNF‐α‐treated NPCs, the cells were transfected with the miR‐21 agonist or antagonist. miR‐21 levels in NPCs were markedly up‐regulated by the miR‐21 agonist and down‐regulated by the miR‐21 antagonist (Fig. 3G). The decreased levels of miR‐21 in TNF‐α‐treated NPCs were rescued by treatment of exosomes from MSCs but not fibroblast. Consistent with the elevated intra‐exosomal miR‐21 levels, TNF‐MSC‐exosomes showed stronger effect on increasing miR‐21 levels in NPCs (Fig. 3H). Overexpression or knockdown of intracellular miR‐21 levels was confirmed in MSC‐exosomes‐treated apoptotic NPCs when exposed to miR‐21‐overexpressing or miR‐21‐deficient exosomes (Fig. 3I), showing that miR‐21 levels in NPCs were changed accordingly with exosomal miR‐21 levels.

### miR‐21 in MSC‐exosomes alleviated TNF‐α induced NPC apoptosis

**Figure 4:**
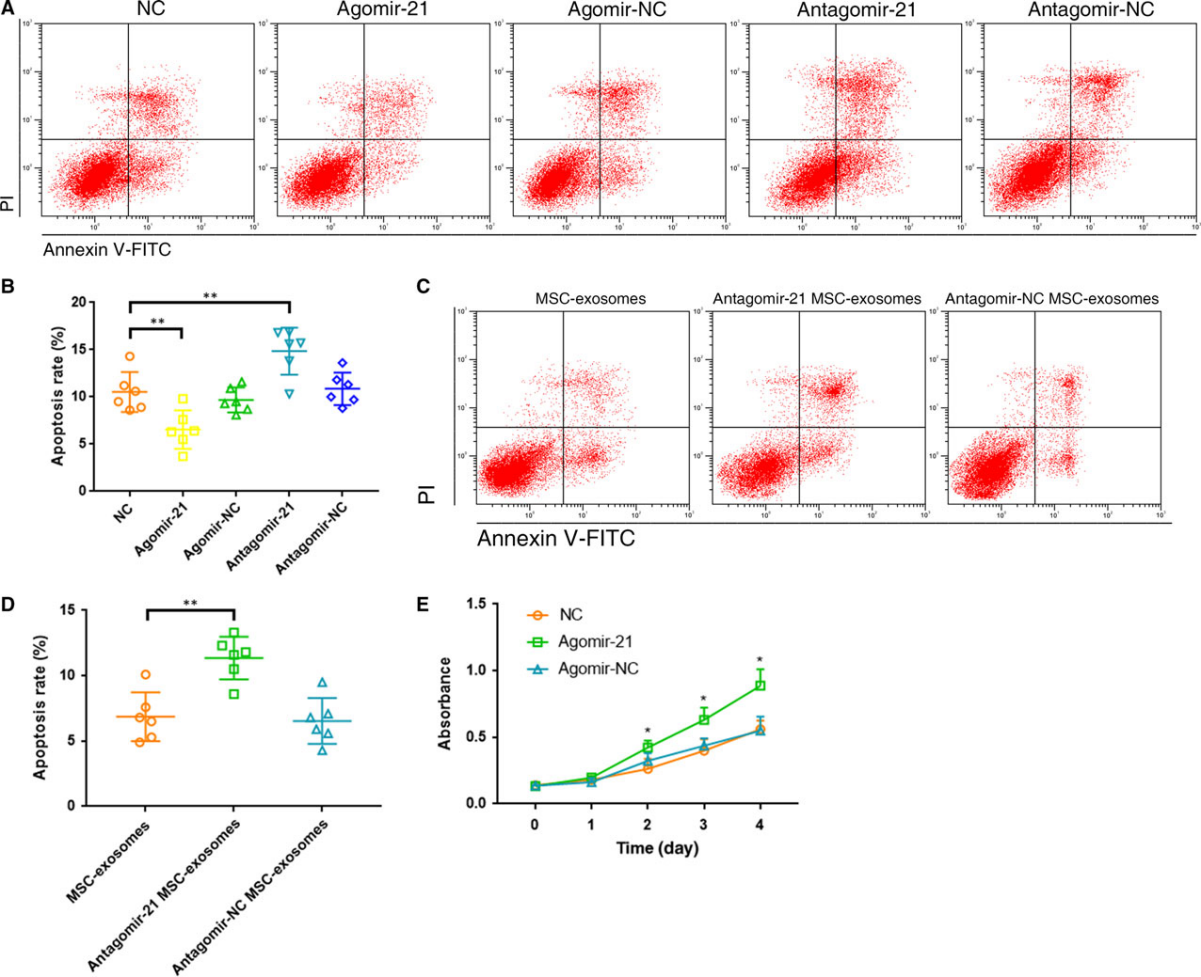
miR‐21 in MSC‐exosomes alleviated TNF‐α induced NPC apoptosis. (**A**) Effect of miR‐21 on NPC apoptosis was evaluated using the flow cytometry analysis. Representative dot plots of cell apoptosis were shown. (**B**) The agomir‐21 significantly alleviated NPC apoptosis (apoptosis rate 6.6% ± 2.0%), while the antagomir‐21 exacerbated apoptosis (apoptosis rate 14.9% ± 2.5%; **P < 0.01 by Kruskal–Wallis test). (**C**) Anti‐apoptotic activities of miR‐21‐depleted MSC‐exosomes were detected using the flow cytometry analysis. Representative dot plots of cell apoptosis were shown. (**D**) The percentage of the apoptotic cells was increased after knockdown of miR‐21 in MSC‐exosomes (apoptosis rate 11.4% ± 1.6% *versus* control 6.9% ± 1.9%; **P < 0.01 by Kruskal–Wallis test). (**E**) CCK‐8 proliferation assay showed that cell proliferation was significantly increased in agomir‐21‐transfected NPCs compared with non‐transfected or agomir‐NC‐transfected cells (* *P <* 0.05 *versus* NC or agomir‐NC by Kruskal–Wallis test).

We investigated the role of miR‐21 in TNF‐α induced apoptosis by over‐ and under‐expressing miR‐21 in NPCs. The agomir‐21 significantly alleviated apoptosis, while the antagomir‐21 exacerbated apoptosis (Fig. 4A and B). When isolated from the supernatant of MSCs transfected with antagomir‐21, exosomes lost its anti‐apoptotic effect (Fig. 4C and D). These findings pointed to rescuing the down‐regulated miR‐21 levels as a potential strategy to protect NPCs from apoptosis and gave reason to suspect that miR‐21 mediated some of the therapeutic benefits of MSC‐exosomes. In addition, we examined the effects of miR‐21 expression on NPC proliferation. CCK‐8 proliferation assay showed that cell proliferation was significantly increased in agomir‐21‐transfected cells compared with non‐transfected or agomir‐NC‐transfected cells (Fig. 4E).

### Delivery of miR‐21 in MSC‐exosomes inhibited NPC apoptosis by targeting PTEN through PI3K‐Akt pathway

**Figure 5:**
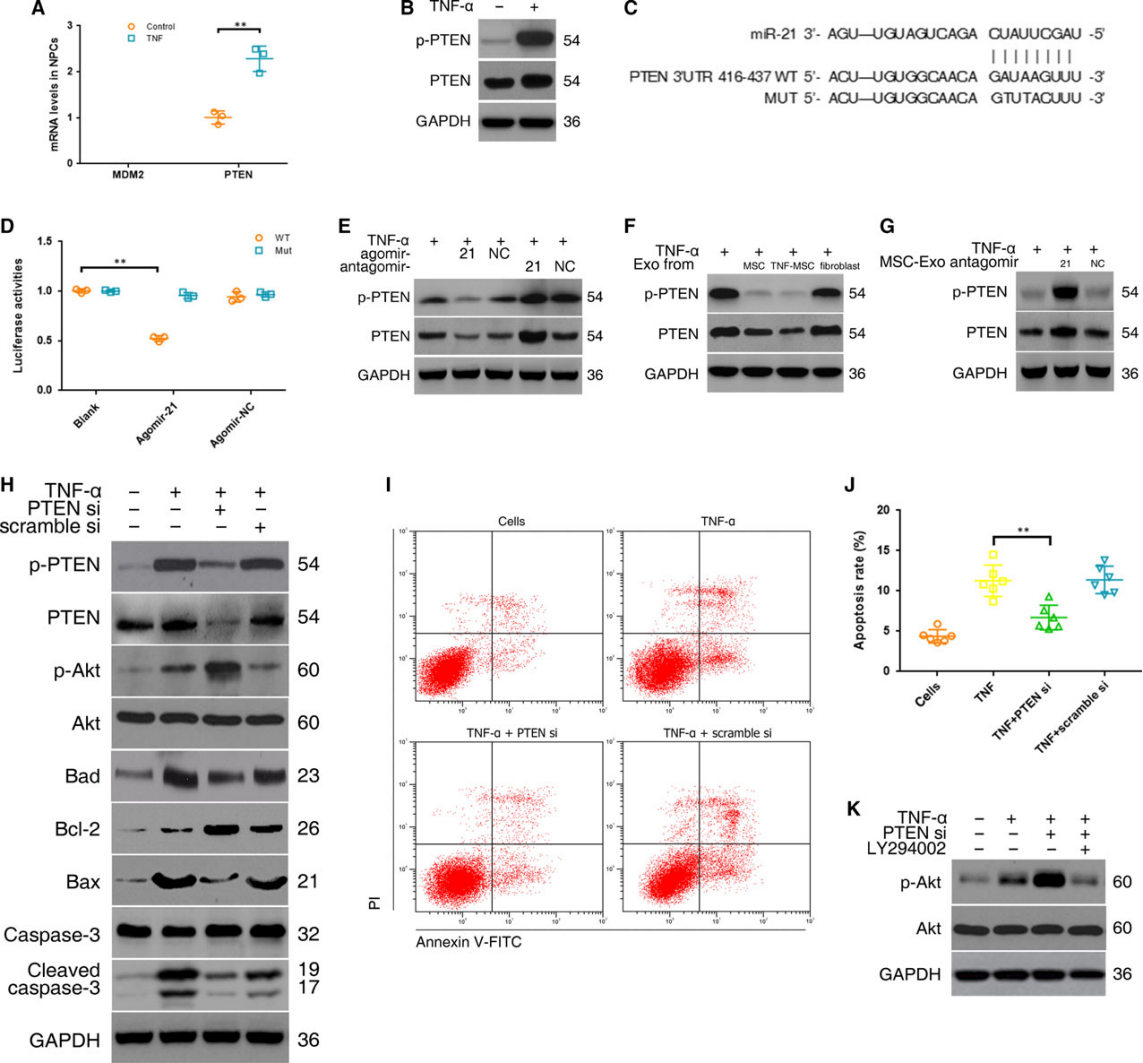
Delivery of miR‐21 in MSC‐exosomes inhibited NPC apoptosis by targeting PTEN through PI3K‐Akt pathway. (**A**) The mRNA expression of MDM2 and PTEN in NPCs was detected using qRT‐PCR. PTEN expression was significantly increased following TNF‐α treatment (*n* = 3; **P < 0.01 by Mann–Whitney *U*‐test). (**B**) Western blot analysed PTEN protein levels in NPCs after TNF‐α treatment. (**C**) 3′‐UTR region of human PTEN mRNA was found to harbour a binding site for hsa‐miR‐21. (**D**) Luciferase reporter assay found that agomir‐21 exclusively decreased luciferase activity of the wild‐type reporter plasmids (*n* = 3; **P < 0.01 by anova test). Fluorescence intensity of firefly and Renilla was (3.80 ± 0.24) × 10^5^ and(7.20 ± 0.83) × 10^6^, respectively, for agomir‐21 cotransfection, and (1.08 ± 0.09) × 10^6^ and(9.48 ± 0.44) × 10^6^, respectively, for agomir‐NC cotransfection. (**E**) Western blot showed that the agomir‐21 significantly reduced while the antagomir‐21 increased the expression levels of PTEN protein compared with the control in TNF‐α‐treated NPCs. (**F**) Western blot showed that MSC‐exosomes (Exo) lowered PTEN protein levels in TNF‐α‐treated NPCs. (**G**) Western blot showed that miR‐21‐deficient MSC‐exosomes lost the ability of MSC‐exosomes to suppress PTEN expression. (**H**) Western blot showed that PTEN‐siRNA (PTEN si) remarkably increased expression of phospho‐Akt and Bcl‐2, and decreased expression of Bad, Bax and cleaved caspase‐3. (**I**) Effect of PTEN‐siRNA on TNF‐α induced NPC apoptosis was evaluated using flow cytometry analysis. Representative dot plots of cell apoptosis were shown. (**J**) PTEN‐siRNA significantly decreased the percentage of the apoptotic cells compared with the control (6.7% ± 1.5% *versus* 11.3% ± 1.9%; **P < 0.01 by Kruskal–Wallis test). (**K**) Western blot showed that the PI3K inhibitor (LY294002) markedly decreased phospho‐Akt protein expression.

To determine the target genes and molecular pathways related to miRNAs in apoptotic NPCs, the common targets and pathways of the five down‐regulated miRNAs induced by TNF‐α were explored by the genes intersection option using the DIANA‐mirPath v3.0 database. The results identified seven validated pathways, five of which were involved in specific cancers and thus eliminated (Table S3). Two remaining signalling pathways (p53 and phosphatidylinositol) and two overlapped target genes (PTEN and murine double minute 2 (MDM2)) were selected for further evaluation. The mRNA expression of MDM2 was not found in NPCs whether the cells were treated with TNF‐α or not. The PTEN mRNA and protein levels in NPCs were significantly increased following TNF‐α treatment (Fig. 5A and B). Further pathway analysis showed that the direct effect of PTEN was inhibition of PI3K/Akt pathway in both p53 and phosphatidylinositol signalling pathway (Fig. S3). The activation of PI3K/Akt pathway is well‐known to provide cells with a survival signal that allows them to withstand apoptotic stimuli. Therefore, PTEN was chosen as the target of miR‐21. 3′‐UTR region of PTEN mRNA was found to harbour a putative binding site that is conserved in different species for miR‐21 using bioinformatics tools (RNAhybrid, miRTarBase, DIANA‐TarBase and PITA) (Fig. 5C, Fig. S4). To verify this finding, the wild‐type or mutant 3′UTR sequence of PTEN gene was cloned into luciferase reporter plasmids. As expected, agomir‐21 exclusively decreased luciferase activity of the wild‐type reporter plasmids, while no suppression of activity was observed with respect to the mutant ones (Fig. 5D). These results demonstrated that the miR‐21 binding site in 3′‐UTR of PTEN mRNA was functional. To further confirm that PTEN is the target of miR‐21 in TNF‐α‐treated NPCs, the levels of PTEN were measured by Western blot. The data showed that the agomir‐21 significantly reduced while the antagomir‐21 increased the expression levels of PTEN protein compared with the control in TNF‐α‐treated NPCs (Fig. 5E). MSC‐exosomes lowered PTEN levels in TNF‐α‐treated NPCs (Fig. 5F). NPCs were incubated with miR‐21‐deficient MSC‐exosomes, and then, the inhibiting effects of MSC‐exosomes on PTEN expression were removed (Fig. 5G), indicating that PTEN was indeed down‐regulated by miR‐21 incorporated in MSC‐exosomes.

The downstream pathway of PTEN in TNF‐α‐treated NPCs was explored after knockdown of PTEN. The PTEN‐siRNA reduced PTEN protein levels compared with the negative control. Moreover, the silencing effects of the PTEN‐siRNA remarkably increased expression of phospho‐Akt and Bcl‐2, and decreased expression of Bad, Bax and cleaved caspase‐3 (Fig. 5H). The relationship between PTEN and NPC apoptosis was detected by flow cytometry analysis. The results showed that PTEN‐siRNA significantly decreased the percentage of the apoptotic cells compared with the control (Fig. 5I and J). Akt activation was suppressed using LY294002 (Fig. 5K), suggesting that Akt was positively regulated by PI3K. Taken together, these results confirmed that the delivery of miR‐21 in MSC‐exosomes attenuated NPC apoptosis by targeting PTEN, probably through PI3K/Akt pathway.

### Intradiscal injection of MSC‐exosomes alleviated the NPC apoptosis and IVD degeneration in a rat model

**Figure 6:**
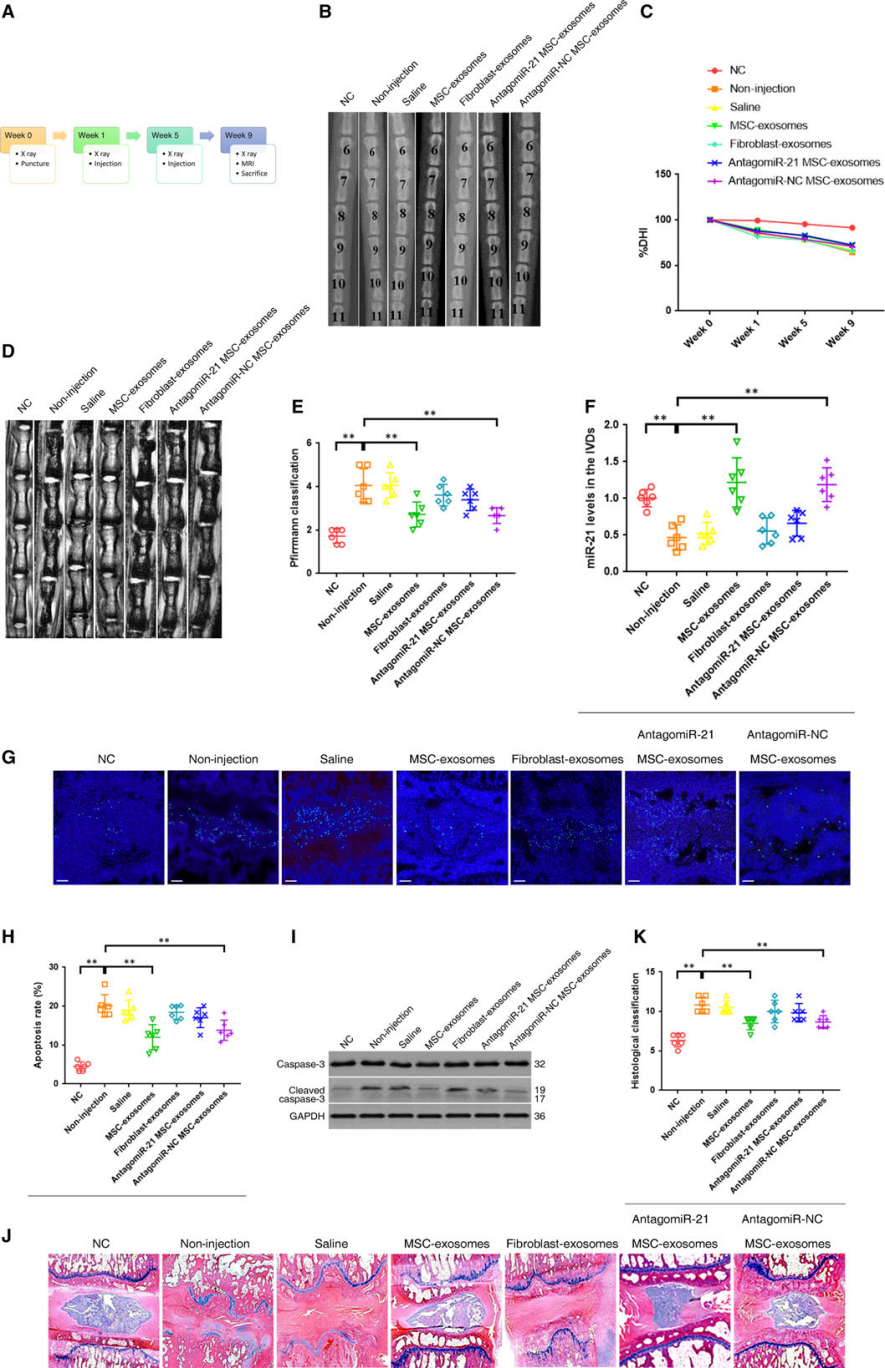
Intradiscal injection of MSC‐exosomes alleviated the NPC apoptosis and IVD degeneration in a rat model. (**A**) A flow diagram of the experiments *in vivo*. (**B**) Radiographs of the indicated groups were obtained 9 weeks after needle puncture. Co6/7, Co8/9 and Co10/11 were punctured with Co7/8 and Co9/10 left intact. (**C**) Changes in disc height index (DHI) of the indicated groups after needle puncture. The DHI was measured at week 0, 1, 5, 9 time point. A significant decrease of the %DHI was observed in all puncture groups at 1 week after surgery (*P* < 0.01by *t* test). At each time point after puncture, a significant decrease of %DHI was noted in all puncture groups compared with the negative control group (*P* < 0.01 by Kruskal–Wallis test). No significant difference was found in the %DHI between all puncture groups. (**D**) MRIs of the indicated groups were obtained 9 weeks after needle puncture. Co6/7, Co8/9 and Co10/11 were punctured with Co7/8 and Co9/10 left intact. (**E**) The change of MRI grade in the indicated groups at 9 weeks after needle puncture. The degree of disc degeneration by MRI grade was significantly lower in the MSC‐exosomes group than in the non‐injection group (**P < 0.01 by Kruskal–Wallis test). (**F**) qRT‐PCR showed that the decreased levels of miR‐21 in the punctured IVDs were rescued by the injection of MSC‐exosomes but not fibroblast‐exosomes (**P < 0.01 by Kruskal–Wallis test). (**G**) TUNEL staining of IVDs in the indicated groups at 9 weeks after needle puncture. Blue fluorescence (DAPI) indicating total cells; Green fluorescence (FITC) indicating TUNEL‐positive cells. Scale bar = 150 μm (**H**) A significant decrease in the apoptosis rate was noted in the MSC‐exosomes group compared with the non‐injection group (**P < 0.01 by Kruskal–Wallis test). (**I**) Western blot analysed the levels of caspase‐3 and cleaved caspase‐3 in the IVDs in the indicated groups at 9 weeks after needle puncture. (**J**) HE staining of IVDs in the indicated groups at 9 weeks after needle puncture. (magnification ×40). (**K**) A significant decrease in the degeneration grade was noted in the MSC‐exosomes group compared with the non‐injection group (** *P <* 0.01 by Kruskal–Wallis test).

We successfully established a rat model of IVD degeneration by the needle puncture (Fig. 6A). At 1 and 5 weeks after the puncture, the MSC‐exosomes were injected into the punctured IVDs using a 33‐gauge fine needle. X‐rays obtained at time 1, 5 and 9 weeks demonstrated progressive disc space narrowing over time in all IVD punctured groups. At each time point after injection, no significant difference in the %DHI was noted between the MSC‐exosomes and non‐injection group (Fig. 6B and C). At 9 weeks after injection, the IVD degeneration score of MRI was significantly lower in the MSC‐exosomes group than in the non‐injection group (Fig. 6D and E). The expression of miR‐21 in the IVDs was remarkably elevated after the injection of MSC‐exosomes (Fig. 6F). For the analysis of apoptosis incidence, there was a significant decrease in the percentage of apoptotic cells and the activation of caspase‐3 in the MSC‐exosomes group compared with the non‐injection group (Fig. 6G, H, I). The histologic score was significantly higher in the non‐injection groups than in the MSC‐exosomes groups at time 9 weeks (Fig. 6J and K). These results revealed the positive effects of MSC‐exosomes injection on reducing the NPC apoptosis and IVD degeneration. However, these effects were markedly suppressed after the down‐regulation of exosomal miR‐21 levels (Fig. 6D–K). No significant difference in any evaluation was observed between the saline injection group and the non‐injection group, indicating a negative effect of the 33‐gauge fine‐needle puncture on the progression of IVD degeneration (Fig. 6B–K). In addition, the injection of fibroblast‐exosomes lacked the ability to inhibit NPC apoptosis and attenuate IVD degeneration (Fig. 6D–K).

## Discussion

Recent evidence suggests that MSCs transplantation into degenerated IVD may be beneficial, and that indirect mechanisms such as paracrine may be partly responsible for the therapeutic benefits. Because exosomes are increasingly emerging as the active component of the paracrine secretion by MSCs, we investigated the protective effects of MSC‐exosomes on TNF‐α induced NPC apoptosis. The results showed that the anti‐apoptotic activities of MSC‐CM were attributed to the release of MSC‐exosomes. In contrast, both MSC‐CM depleted of exosomes and fibroblast‐exosomes lacked the anti‐apoptotic activities, suggesting the negligible roles for extracellular soluble factors secreted by MSCs and the importance of exosomes parent cells. MSCs are ideal parent cells for exosomes therapy, because they are easily available and expansible from diverse tissues, prolific producer of exosomes, and proven to be clinically safe and not immunologically reactive 34. MSC‐exosomes confer the similar benefits as MSCs by delivering exogenous therapeutic agents particularly miRNAs in many diseases and are regarded as cell‐free therapeutic candidates 20, 35, 36.

miRNAs, as the crucial mediators for the benefits of MSC‐exosomes, can provide sustained therapeutic effect and fundamental alterations of the local microenvironment 37. Recently, many studies have reported that different miRNAs regulate NPC apoptosis through their targets in the degenerated IVD. Pro‐apoptotic miRNAs include miR‐494, miR‐138, miR‐27a and miR‐15a 5, 23, 24, 25, and anti‐apoptotic miRNAs include miR‐98, miR‐155 and miR‐125a 26, 27, 28. However, little is known about which miRNAs are associated with TNF‐α induced NPC apoptosis. As the supplement of MSC‐exosomes could mitigate NPC apoptosis and MSC‐exosomes have been proved to be enriched in miRNAs, it seems reasonable to speculate that the potentially effective miRNAs as connectors should be down‐regulated in apoptotic NPCs and abundant in MSC‐exosomes. Therefore, differentially expressed miRNAs in TNF‐α induced apoptotic NPCs were detected by the microarray chip analysis, and then, five down‐regulated miRNAs were confirmed by qRT‐PCR, suggesting a possible connection between the decreases of these miRNAs and the NPC apoptosis. Among these miRNAs, only miR‐21 levels in MSC‐exosomes were significantly greater than that in fibroblasts‐exosomes, providing us with a potential mediator of the TNF‐α induced NPC apoptosis and the anti‐apoptotic effect of MSC‐exosomes.

Exosomal miR‐21 levels were elevated and reduced by transfecting exosome‐producing MSCs with the miR‐21 agonist and antagonist, respectively. Combined with the observations that miR‐21 within exosomes was protected from RNase degradation and that Ct value of miR‐21 was lower in exosomes, all of these results reflected that MSC‐exosomes were enriched in miR‐21. When incubated with MSC‐exosomes, NPCs could take up the exosomes, and the exosomal miR‐21 could be internalized into the recipient NPCs. In addition, MSC‐exosomes containing abundant miR‐21 could increase the concentration of miR‐21 in apoptotic NPCs. In the TNF‐α‐treated NPCs, the miR‐21 agonist up‐regulated miR‐21 expression and effectively promoted cell survival, whereas the miR‐21 antagonist down‐regulated miR‐21 expression and enhanced cell apoptosis. MSC‐exosomes with down‐regulated miR‐21 levels lost the capacity to attenuate apoptosis. Collectively, these findings confirmed the function of miR‐21 in protecting NPCs from TNF‐α‐induced apoptosis and mediating the efficacies of MSC‐exosomes against apoptosis.

A previous paper has reported that miR‐21 up‐regulation could be observed in NP tissues obtained from patients with IVD degeneration and contribute to abnormal NPC proliferation 38. This result seems not in line with our data showing down‐regulated miR‐21 in apoptotic NPCs. The donors of the NP tissues in that previous study were patients undergoing surgical treatment, implying that their IVD degeneration should be in advanced or later stages. By contrast, NPCs were harvested from patients without IVD degeneration in this study. Several previous studies have proved that TNF‐α could suppress miR‐21 expression in early stages at the cellular level 39, 40. On the other hand, TNF family members, including TNF‐α and TNF‐related apoptosis‐inducing ligand, could up‐regulate miR‐21 levels only in later stages to induce apoptosis‐resistant or anti‐inflammatory responses 41, 42. Our results also proved that miR‐21 promoted NPC proliferation. Given that miR‐21 has been shown to regulate cell survival by promoting proliferation and inhibiting apoptosis in different cell types 43, the differences of miR‐21 levels may be explained by the concept that TNF‐α depressed miR‐21 expression during early stages to induce apoptosis and conversely activated miR‐21 expression in later stages to cause apoptosis‐resistant or cell proliferation in NPCs. Moreover, the elevated miR‐21 levels in the degenerated IVD may also be attributed to the supplementation of exosomes secreted from recruited endogenous MSCs or other stem cells for self‐healing 44.

We further investigated the function of MSC‐exosomes and miR‐21 in a rat model of IVD degeneration. Consistent with the results from *in vitro* studies, the effects of miR‐21 in MSC‐exosomes on inhibition of NPC apoptosis were proved by *in vivo* experiments. In addition, the delivery of exosomal miR‐21 could attenuate or delay the IVD degeneration by inhibiting NPC apoptosis in the rat model. Although the injection of MSC‐exosomes did not restore the disc height as expected, the conclusion could be drawn from the results of MRI assessment and histologic examinations.

TNF‐α plays a vital role in the pathological process of IVD degeneration. Numerous pathogenic factors of IVD degeneration, such as aging, smoking and abnormal biomechanical loading, could contribute to TNF‐α concentration increases 2. Elevated TNF‐α can activate the effector caspases (*e.g*. caspase‐3) to exert apoptotic effects *via* the extrinsic and intrinsic pathways. Therefore, we selected TNF‐α as the agent to induce NPC apoptosis. During IVD degeneration, TNF‐α is expressed in the NP tissue of humans and animal models. Additionally, TNF‐α is not only produced by leucocytes, but also by NPCs themselves 3. For this reason, transplanted MSCs are stayed in an inflammatory environment. In this study, a group of MSCs was pretreated with TNF‐α to mimic the inflammatory environment in the degenerated IVD. These MSCs showed increased secretion of exosomes, elevated contents of exosomal miR‐21 and enhanced anti‐apoptotic activities. Previous studies have implied that pretreatment of MSCs with TNF‐α stimulates cell proliferation and enhances therapeutic effects 45, 46. Furthermore, TNF‐α stimulation strengthens the ability of MSCs to release contents within secretory vesicles 47, 48. It seems that the inflammatory environment or pretreatment with TNF‐α could enhance the anti‐apoptotic function of MSCs because of increased exosomes secretion.

Usually miRNAs mediate cell function by inhibiting the post‐transcription process of downstream target genes. The present results demonstrated decreased levels of five miRNAs in TNF‐α‐induced apoptotic NPCs and indicated PTEN as the common target of these miRNAs. Although TNF‐α as a widely used inducer of apoptosis can lead to cell death through several pathways, our results implied that PTEN was critical in NPC apoptosis associated with TNF‐α and miRNAs. The anti‐apoptotic effect of miR‐21 is associated with functional down‐regulation of multiple target genes, such as PTEN, Apaf‐1, DR‐5 and PDCD4 43, 49, 50, all of which are known as pro‐apoptotic genes. In our study, the luciferase reporter and Western blot analysis confirmed that PTEN was the direct target of miR‐21 and thus negatively regulated by miR‐21 in TNF‐α‐treated NPCs. Moreover, MSC‐exosomes successfully down‐regulated PTEN in NPCs, indicating that both endogenous and exogenous miR‐21 functioned similarly.

PTEN silencing caused a series of downstream apoptosis‐related responses in TNF‐α‐treated NPCs, including increased activation of Akt and Bcl‐2, and decreased activation of Bad, Bax and caspase‐3, and eventually inhibited apoptosis. Despite repressing PTEN expression, the activation of Akt was still suppressed using the PI3K inhibitor, confirming that PTEN was a negative regulator of PI3K/Akt pathway. The observation is in accordance with previous reports indicating that the pro‐apoptotic effect of PTEN is through disrupting PI3K/Akt pathway in other cell types 51. All things considered, these findings strongly supported that PTEN‐PI3K‐Akt pathway was the underlying mechanism of miR‐21‐mediated apoptosis protection in NPCs.

The use of MSC‐exosomes as a cell‐free products offers several advantages compared to MSCs, such as reduced risk of iatrogenic tumour formation, malformations or microinfarctions, more convenient manufacture and storage, more stable and sustained biological activity, and minimal immunogenicity allowing allogeneic transplantation 52. MSC‐exosomes may have a potential for circumventing some limitation of using MSCs as therapeutic agents for the treatment of NPC apoptosis and IVD degeneration. The conventional way for MSCs to access the NP tissue is although the AF by needle puncture. However, needle puncture will trigger lesions of the IVD and induce development or progression of IVD degeneration, which has been verified by animal models and clinical studies 53. When using a needle with a larger diameter, greater penetration force is required and will result in more severe tearing of the AF, probably causing accelerated IVD degeneration or leakage of transplanted cells. In this study, a 33‐gauge diameter needle was selected for injection because it would not exacerbate further IVD degeneration in the rat model 54. Our results indeed confirmed that the 33‐gauge needle and the effect of injecting a small volume of saline did not accelerate IVD degeneration. Smaller needle diameters facilitate minimizing the IVD lesion, but could compromise transplanted cell viability through direct shear forces 55. Exosomes as nano‐sized vesicles could be delivered using a needle as small as possible to ensure negligible effect on IVD integrity. Meanwhile, its biological activity would not be affected by the increased inner pressure of the needle. Furthermore, exosomes has a potential to infiltrate into the NP through the endplate, providing some possible delivery strategies such as injection *via* pedicle or venous, to keep the AF intact 56, 57. Thus, MSC‐exosomes therapy may be a safer and a more effective modality than cell‐based MSC therapy for the treatment of NPC apoptosis and IVD degeneration.

Although our work implicated MSC‐exosomes and exosomal miR‐21mediating the anti‐apoptotic effect in NPCs, it remains unclear whether other extracellular vesicles or exosomal cargoes function as similar roles. It awaits further investigations.

## Conclusion

**Figure 7:**
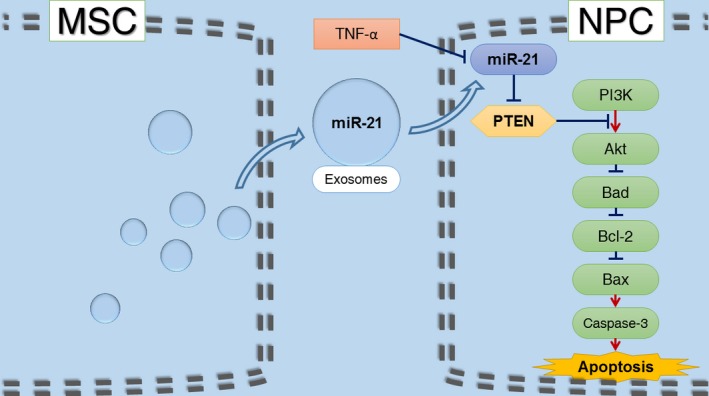
Schematic of our working hypothesis. MSC‐derived exosomes prevent NPCs from TNF‐α‐induced apoptotic process *via* miR‐21 contained in exosomes. Exosomal miR‐21 restrains PTEN and thus activates PI3K/Akt pathway in apoptotic NPCs.

In conclusion, MSC‐derived exosomes prevent NPCs from apoptotic process and alleviate IVD degeneration, at least partly, *via* miR‐21 contained in exosomes. Exosomal miR‐21 restrains PTEN and thus activates PI3K/Akt pathway in apoptotic NPCs (Fig. 7). Our work confers a promising therapeutic strategy for IVD degeneration.

## Conflicts of interest

The authors confirm that there are no conflicts of interest.

## Supporting information


**Figure S1** MSCs were characterized by the expression of CD73, CD90 and CD105 and lack of expression of CD34 and CD45 surface molecules using flow cytometry.

**Figure S2** Heat map of the miRNA microarray expression data from TNF‐α treated and non‐treated NPCs. Hierarchical clustering results of differentially‐expressed miRNAs.

**Figure S3** KEGG pathway showed that the direct effect of PTEN was inhibition of PI3K/Akt pathway in both p53 and phosphatidylinositol signaling pathway.

**Figure S4** 3′‐UTR region of PTEN mRNA was found to harbor a putative binding site that is conserved in different species for miR‐21.

**Table S1** Sequence used in this study.

**Table S2** All the differentially expressed miRNAs in TNF‐α treated NPCs and untreated controls.

**Table S3** Pathways and genes related to TNF‐α induced downregulation of miRNAs in NPCs.
