# Supplementary figures and images for: Mesenchymal stem cells deliver exogenous miR‐21 via exosomes to inhibit nucleus pulposus cell apoptosis and reduce intervertebral disc degeneration
Source: J Cell Mol Med. 2017 Aug 14;22(1):261–76. doi: 10.1111/jcmm.13316 (PMC5742691; doi:10.1111/jcmm.13316)

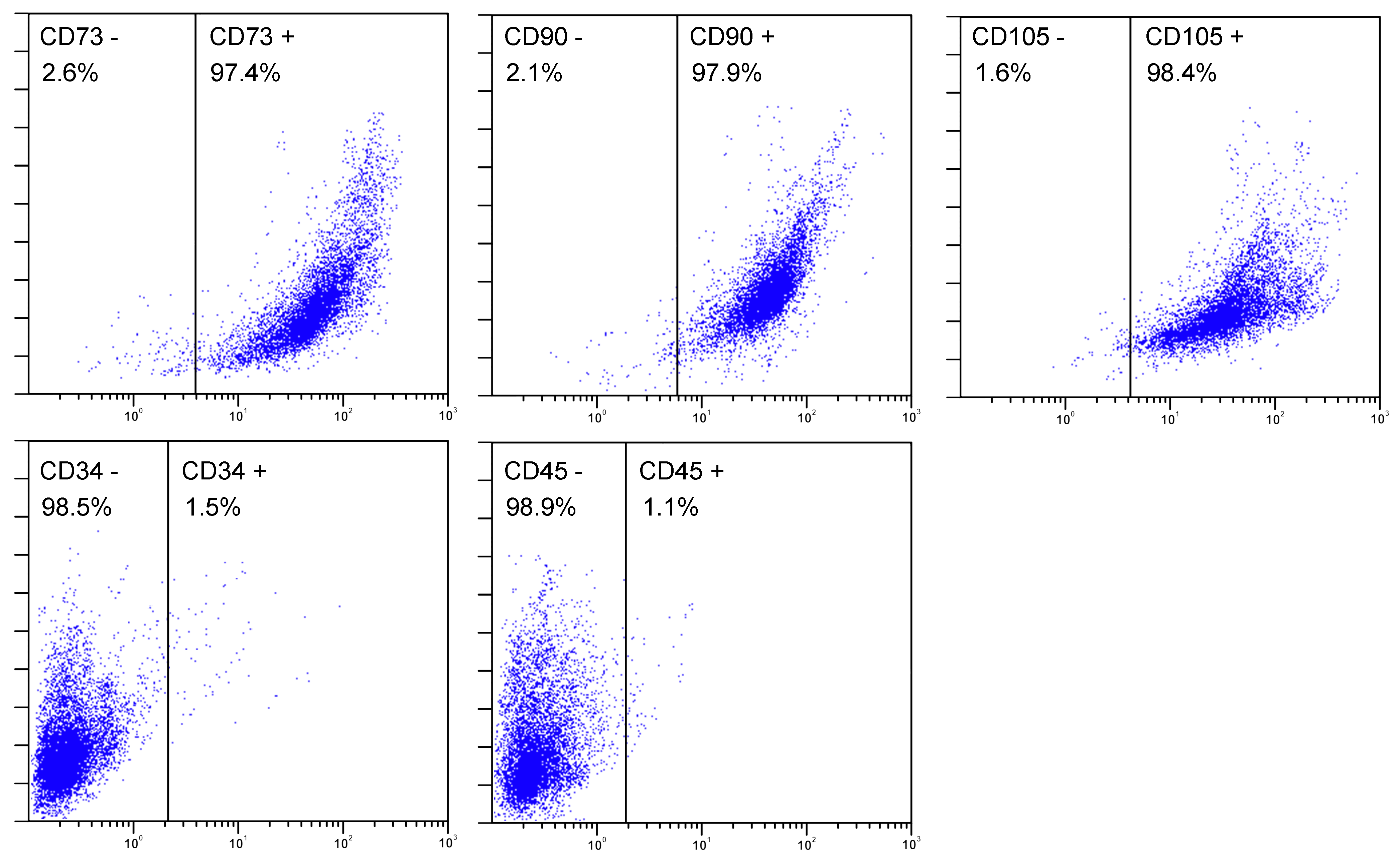

Supplement: Supplementary file 1 — Figure S1 MSCs were characterized by the expression of CD73, CD90 and CD105 and lack of expression of CD34 and CD45 surface molecules using flow cytometry. [file JCMM-22-261-s001.tiff]

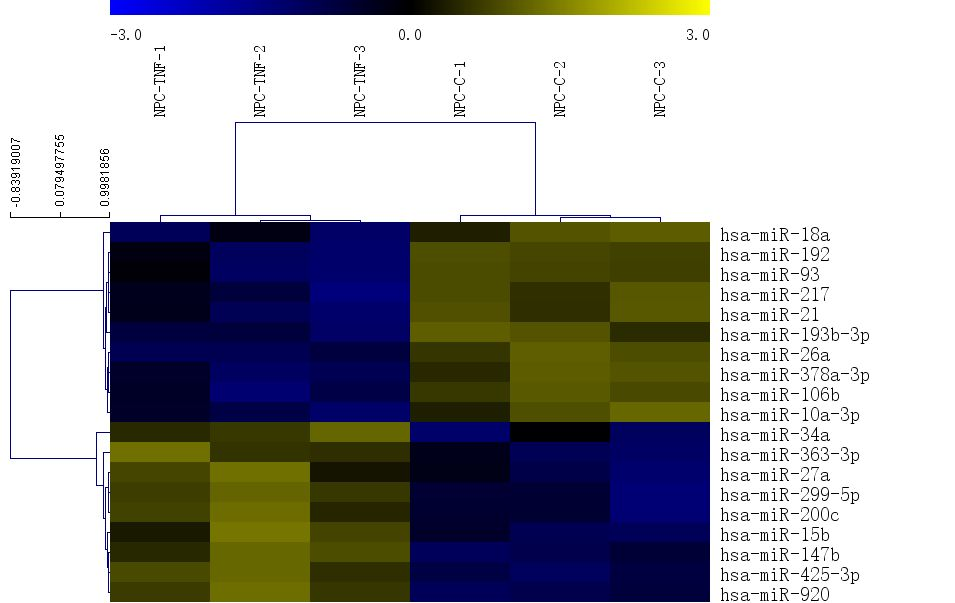

Supplement: Supplementary file 2 — Figure S2 Heat map of the miRNA microarray expression data from TNF‐α treated and non‐treated NPCs. Hierarchical clustering results of differentially‐expressed miRNAs. [file JCMM-22-261-s002.tiff]

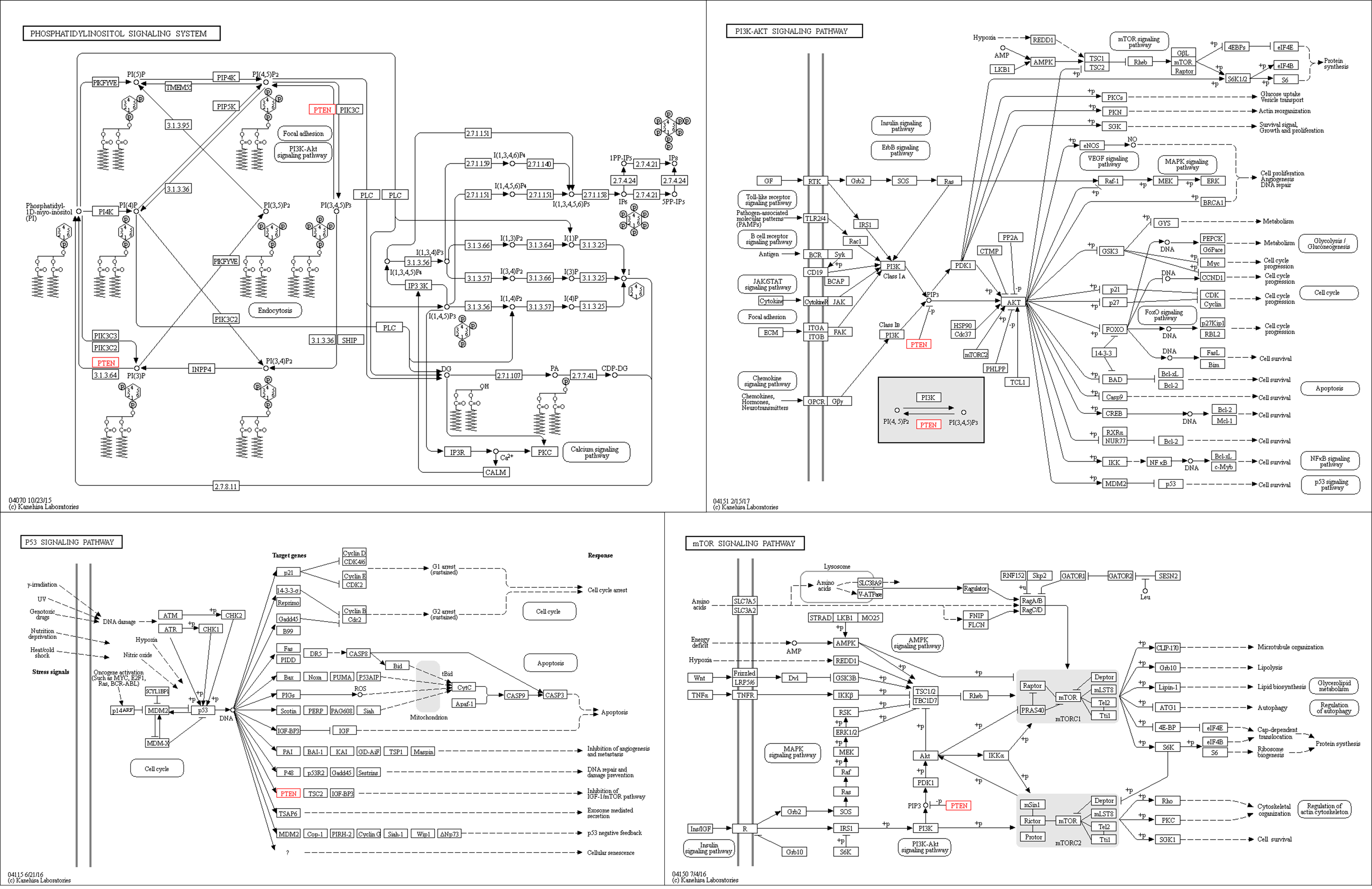

Supplement: Supplementary file 3 — Figure S3 KEGG pathway showed that the direct effect of PTEN was inhibition of PI3K/Akt pathway in both p53 and phosphatidylinositol signaling pathway. [file JCMM-22-261-s003.tiff]

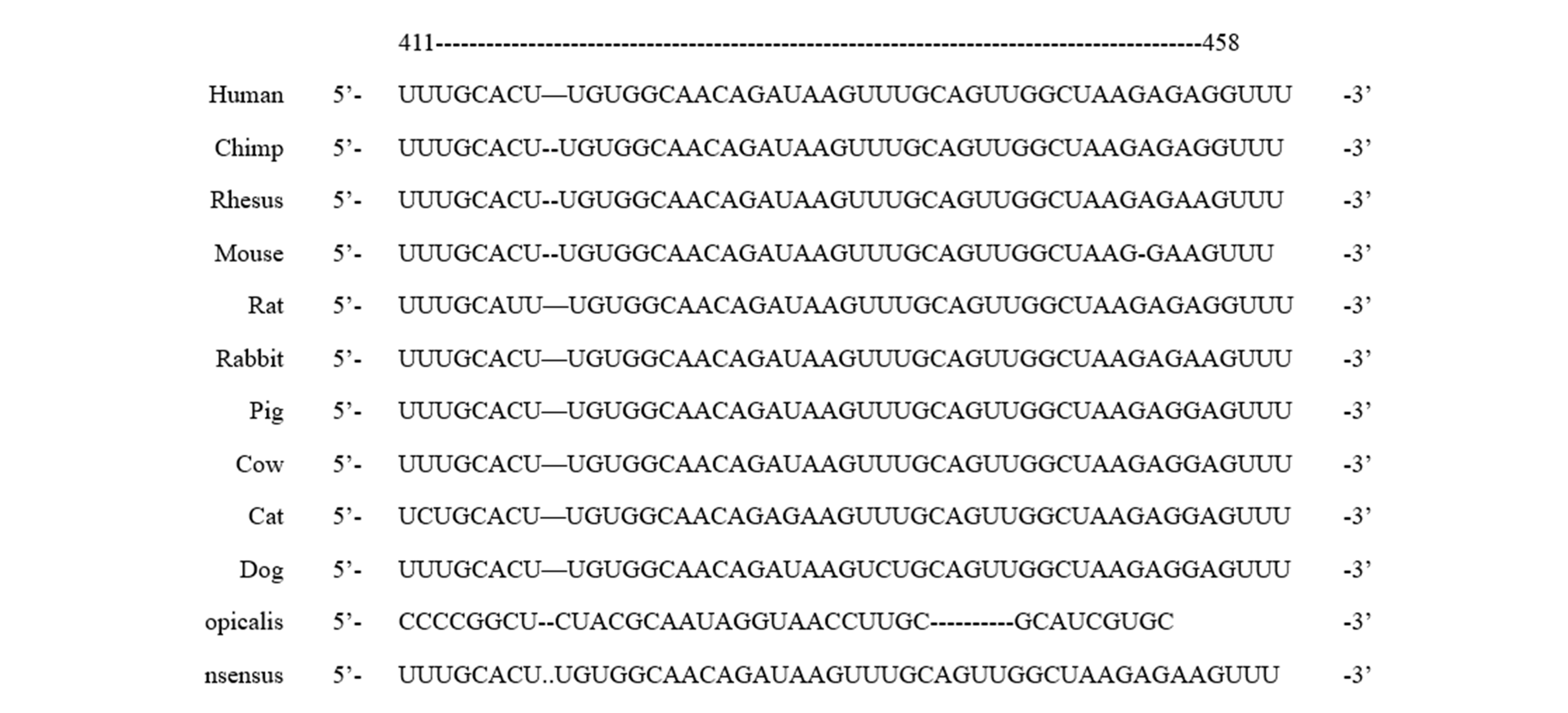

Supplement: Supplementary file 4 — Figure S4 3′‐UTR region of PTEN mRNA was found to harbor a putative binding site that is conserved in different species for miR‐21. [file JCMM-22-261-s004.tiff]
