# Supplementary material for: Mesenchymal stem cells deliver exogenous miR‐21 via exosomes to inhibit nucleus pulposus cell apoptosis and reduce intervertebral disc degeneration
Source: J Cell Mol Med. 2017 Aug 14;22(1):261–76. doi: 10.1111/jcmm.13316 (PMC5742691; doi:10.1111/jcmm.13316)
Supplement: Supplementary file 5 — Table S1 Sequence used in this study. [file JCMM-22-261-s005.docx]

Supplementary Table 1 Sequence used in this study

|  |  | Sequence |
| --- | --- | --- |
| agomir-21 |  | UAGCUUAUCAGACUGAUGUUGA |
| agomir-NC |  | UUUGUACUACACAAAAGUACUG |
| antagomir-21 |  | UGUCGGGUAGCUGACCACAAC |
| antagomir-NC |  | AAACAUGAUGUGUUUUCAUGAC |
| PTEN siRNA | Sense | CGCGTCCCCGCCAAATTTAACTGCAGAGTTCAAGAGACTCTGCAGTTAAATTTGGCTTTTTGGAAAT |
|  | Antisense | CGATTTCCAAAAAGCCAAATTTAACTGCAGAGTCTCTTGAACTCTGCAGTTAAATTTGGCGGGGA |
| Scramble siRNA | Sense | CGUACUGUCGACACUGAAACGGACA |
|  | Antisense | UAUCCGUUUCAGUGUCGACAGUACGTG |
| PTEN-3'UTR | Forward | CGATTCTAGAAATCATGTTCTGGTGG |
|  | Reverse | GCATTCTAGAATTCTGCACAGTAAGCATA |
