# Supplementary material for: Mesenchymal stem cells deliver exogenous miR‐21 via exosomes to inhibit nucleus pulposus cell apoptosis and reduce intervertebral disc degeneration
Source: J Cell Mol Med. 2017 Aug 14;22(1):261–76. doi: 10.1111/jcmm.13316 (PMC5742691; doi:10.1111/jcmm.13316)
Supplement: Supplementary file 6 — Table S2 All the differentially expressed miRNAs in TNF‐α treated NPCs and untreated controls. [file JCMM-22-261-s006.docx]

Supplementary Table 2 All the differentially expressed miRNAs in TNF-α treated NPCs and untreated controls

|  |  | *P* | Fold change |
| --- | --- | --- | --- |
| Downregulation | hsa-miR-192 | 6.250E-04 | 0.10 |
|  | hsa-miR-26a | 6.704E-03 | 0.10 |
|  | hsa-miR-93 | 7.879E-04 | 0.12 |
|  | hsa-miR-217 | 5.881E-03 | 0.17 |
|  | hsa-miR-21 | 5.319E-03 | 0.21 |
|  | hsa-miR-106b | 2.320E-03 | 0.22 |
|  | hsa-miR-378a-3p | 7.068E-03 | 0.25 |
|  | hsa-miR-193b-3p | 3.936E-03 | 0.34 |
|  | hsa-miR-18a | 1.316E-02 | 0.35 |
|  | hsa-miR-10a-3p | 1.213E-02 | 0.40 |
| Upregulation | hsa-miR-15b | 1.973E-02 | 2.03 |
|  | hsa-miR-299-5p | 4.919E-03 | 2.67 |
|  | hsa-miR-425-3p | 4.679E-03 | 2.71 |
|  | hsa-miR-34a | 2.462E-02 | 2.75 |
|  | hsa-miR-27a | 3.548E-02 | 2.77 |
|  | hsa-miR-363-3p | 2.361E-02 | 3.07 |
|  | hsa-miR-147b | 1.001E-02 | 3.75 |
|  | hsa-miR-200c | 2.353E-02 | 4.19 |
|  | hsa-miR-920 | 2.642E-02 | 7.89 |
