# Supplementary material for: Mesenchymal stem cells deliver exogenous miR‐21 via exosomes to inhibit nucleus pulposus cell apoptosis and reduce intervertebral disc degeneration
Source: J Cell Mol Med. 2017 Aug 14;22(1):261–76. doi: 10.1111/jcmm.13316 (PMC5742691; doi:10.1111/jcmm.13316)
Supplement: Supplementary file 7 — Table S3 Pathways and genes related to TNF‐α induced downregulation of miRNAs in NPCs. [file JCMM-22-261-s007.docx]

Supplementary Table 3

Pathways and genes related to TNF-α induced downregulation of miRNAs in NPCs

|  | KEGG pathway | *P* value | Genes |
| --- | --- | --- | --- |
| 1 | MicroRNAs in cancer (hsa05206) | 1.6699189084e-06 | DICER1, PTEN, MDM2 |
| 2 | p53 signaling pathway (hsa04115) | 0.00260960359675 | PTEN, MDM2 |
| 3 | Phosphatidylinositol signaling system (hsa04070) | 0.00649418437377 | PTEN |
| 4 | Glioma (hsa05214) | 0.00649418437377 | PTEN, MDM2 |
| 5 | Melanoma (hsa05218) | 0.0304086704662 | PTEN, MDM2 |
| 6 | Chronic myeloid leukemia (hsa05220) | 0.0304086704662 | MDM2 |
| 7 | Prostate cancer (hsa05215) | 0.0333265363486 | PTEN, MDM2 |
