# Supplementary material for: Mesenchymal stem cells deliver exogenous miR‐21 via exosomes to inhibit nucleus pulposus cell apoptosis and reduce intervertebral disc degeneration
Source: J Cell Mol Med. 2017 Aug 14;22(1):261–76. doi: 10.1111/jcmm.13316 (PMC5742691; doi:10.1111/jcmm.13316)
Supplement: Supplementary file 8 [file JCMM-22-261-s008.docx]

Supplementary Figure 1

MSCs were characterized by the expression of CD73, CD90 and CD105 and lack of expression of CD34 and CD45 surface molecules using flow cytometry.

Supplementary Figure 2

Heat map of the miRNA microarray expression data from TNF-α treated and non-treated NPCs. Hierarchical clustering results of differentially-expressed miRNAs. Each row represents the expression profile of a miRNA across 6 samples and each column represents a sample. Yellow and blue colors indicate higher and lower expression levels of the miRNAs, respectively.

Supplementary Figure 3

KEGG pathway showed that the direct effect of PTEN was inhibition of PI3K/Akt pathway in both p53 and phosphatidylinositol signaling pathway.

Supplementary Figure 4

3′-UTR region of PTEN mRNA was found to harbor a putative binding site that is conserved in different species for miR-21.
